# A Parent-Facing Decision Aid About Childhood Vaccination: Co-development Study Adapting a Shared Decision-Making Approach to Support Evidence-Guided Decision-Making

**DOI:** 10.2196/90729

**Published:** 2026-09-02

**Authors:** Daphne Bussink-Voorend, Lisa Vandeberg, Jeannine LA Hautvast, Glyn Elwyn, Marlies EJL Hulscher

**Affiliations:** 1 Primary and Community Care Radboud Institute for Medical Innovation Radboud University Medical Center Nijmegen The Netherlands; 2 Behavioural Science Institute Radboud University Nijmegen The Netherlands; 3 Dartmouth Institute for Health Policy and Clinical Practice Dartmouth College Hanover, NH United States; 4 IQ Health Science Department Radboud Institute for Medical Innovation Radboud University Medical Center Nijmegen The Netherlands

**Keywords:** routine childhood vaccination, decision-making, vaccine hesitancy, user-centered design, decision aid

## Abstract

**Background:**

Childhood vaccination rates are declining, and more parents are becoming hesitant about vaccinating their children. Higher levels of vaccine hesitancy occur among parents with lower levels of educational attainment, who are also likely to have lower levels of health literacy.

**Objective:**

We aimed to develop a web-based decision aid, considered a shared decision-making (SDM) approach, to inform and support Dutch parents in their decision-making about routine childhood vaccination, prioritizing the needs of hesitant parents and those with lower health literacy. We adapted the traditional SDM approach by balancing the ethical tension between the collective responsibility for public health and individual autonomy in vaccination decisions and focused the decision aid on evidence-guided (rather than preference-sensitive) decision-making.

**Methods:**

The development process involved content development and web application development. We adopted a user-centered approach by actively engaging stakeholders, including parents, health professionals, and other experts. Participating stakeholders were purposefully selected and engaged through different routes to include a variety of perspectives. The general concept of vaccination as an evidence-based intervention to promote health at the individual and societal levels remained central throughout the development process.

**Results:**

We conducted this project in the Netherlands from early 2023 through the end of 2024. We identified parental needs through interviews with 18 parents and a literature assessment. Parental needs, evidence-based information about vaccination, and how to communicate about vaccination informed the initial content of the decision aid. After feedback from the advisory group and subject matter experts, we refined the draft. At this stage, the textual content was inserted into a prototype web application. We subjected this prototype to 3 iterative cycles of testing, review, and adaptation. We analyzed and discussed the issues identified during testing and recommended adaptations to improve the design, structure, and content of the decision aid. These adaptations were subsequently discussed with the advisory group before being implemented. In response to the issues raised, we shortened the text blocks, replaced written information with visual elements, and restructured the layout to improve navigation. The final prototype consisted of the modules “Information” and “Decision Support.”

**Conclusions:**

We co-developed a parent-facing decision aid (PDA) for childhood vaccination tailored to the needs of hesitant parents and those with lower health literacy. Through this intervention, we adapted an SDM approach to evidence-guided decision-making, creating space for individual concerns and deliberations without compromising evidence-based recommendations. Future research should focus on the timing of the intervention and the optimal window for offering the PDA in relation to the parents’ decision-making process.

## Introduction

In March 2025, the World Health Organization (WHO) reported the highest number of measles cases in more than 25 years [1]. This increase is attributed to falling vaccination coverage rates, which are inadequate to reach group immunity. Lower public confidence in the safety and necessity of vaccination has led to vaccine hesitancy and decreasing uptake [2]. This poses a significant public health concern because individual decisions to decline vaccination have a broader impact by reducing group immunity [3]. In particular, vulnerable groups, such as very young or ill children, benefit from group immunity for their protection. Promoting these societal aspects of vaccination when discussing individual vaccination decisions may contribute to closing these immunity gaps [4,5]. Besides societal aspects, it is also important to consider cognitive and emotional aspects when developing strategies to address vaccine hesitancy [6].

Decision aids (DAs) are tools developed to support health decisions about prevention, screening, and treatment. When using these tools, people are better informed and more aware of their personal risks and preferences [7]. Vaccination-specific DAs support an active role in decision-making, increase vaccine uptake, and are recommended as interventions to address vaccine hesitancy [8,9]. So far, DAs for vaccination have primarily focused on single vaccinations [10]. However, childhood vaccinations are typically offered as a series of combined vaccines using a timetabled schedule rather than being offered on a one-by-one basis. Yet, few DAs address childhood vaccination as a general concept. Therefore, there is a mismatch between these DAs and the reality of how childhood vaccination is organized and delivered. We considered this an opportunity to develop a web-based parent-facing DA (PDA) that covers all vaccinations offered through the Dutch National Immunization Program (NIP) to children aged ≤4 years.

DAs are originally designed to support shared decision-making (SDM), a collaborative process between patients and health care professionals to reach evidence-based and value-aligned decisions [11]. Typically, DAs present different options in a balanced manner without steering users toward a predetermined outcome, which is suitable for preference-sensitive decisions that primarily affect the individual [12]. However, this approach does not align with the context of routine childhood vaccination, where there is a strong public health imperative. Vaccination is a public health intervention for which the evidence of benefits outweighs the evidence of risks for both individuals and populations. We therefore argue that vaccination decisions, as they are not neutral from a public health perspective, should be evidence-guided rather than focused on individual preferences. This fundamental difference should be reflected in DAs about vaccination by presenting evidence-based individual- and population-level information while allowing space to explore individual concerns and deliberations without compromising evidence-guided decision-making [13].

Since the COVID-19 pandemic, parents have become increasingly concerned about vaccinating their children [14]. In the Dutch context, 27% of parents report hesitancy about their vaccination decisions [15]. Given these rising levels of vaccine hesitancy, parents might benefit from a DA about childhood vaccination. As vaccine hesitancy levels are higher among parents with lower levels of educational attainment and lower health literacy, we aimed to tailor the DA to this group [16,17]. To achieve this, we applied a user-centered approach by involving relevant stakeholders throughout the development of the PDA.

We developed a PDA to support informed and deliberate decision-making about childhood vaccination for Dutch parents. Because decision-making about vaccination is likely to start before a baby is born, we also considered prospective parents to be part of the target group [18]. In this article, we describe the evidence base, guiding principles, stakeholder involvement, and the various steps in the development of this PDA, following the Guidance for the Reporting of Intervention Development (GUIDED) framework [19].

## Methods

### Context of the Intervention

The Dutch NIP is coordinated by the National Institute of Public Health in the Netherlands and executed by nurses and clinicians working at community-based preventive child and youth health care (CYH) centers. Vaccination services are integrated with routine health visits from childbirth onward at CYH centers to monitor growth and development from birth. Up to 95% of parents attend routine CYH center visits. Other health care professionals, such as midwives or general practitioners, are not involved in providing education or counseling about childhood vaccination.

### Purpose and Target Population

We aimed to develop the PDA to support parents in making informed and deliberate decisions about childhood vaccination. The target group consisted of prospective parents and parents of children aged ≤4 years. We targeted this broad and heterogeneous group with a particular focus on those who were hesitant or had lower levels of educational attainment.

### Guiding Principles

The first guiding principle in the development of this intervention was to address vaccination as a public health approach. We aimed to convey accurate scientific knowledge about the role of vaccination in preventing diseases for individuals and across populations. A range of stakeholders guided us in determining how best to present these concepts to vaccine-hesitant parents.

A second principle was to address childhood vaccination as a general concept rather than focusing on single vaccines. We described childhood vaccination as a timed schedule of single-disease and combined vaccines, as offered in the Netherlands.

The third principle was to consider parents’ wider needs beyond information. Other factors such as personal values and others’ experiences and advice play a role in vaccination decisions [20,21].

As a final principle, we prioritized the needs of hesitant parents or those with lower health literacy skills when faced with conflicting advice from stakeholders.

### Use of Components From an Existing Intervention

For our proposed PDA, we adapted a software template previously developed for a DA on human papillomavirus vaccination [22]. The template has also been used to develop other DAs for vaccination [23,24] and allowed us to make adjustments, redesign the layout, and remove or add features.

### Development Approach and Stakeholder Contributions

#### Overview

**Figure 1:**
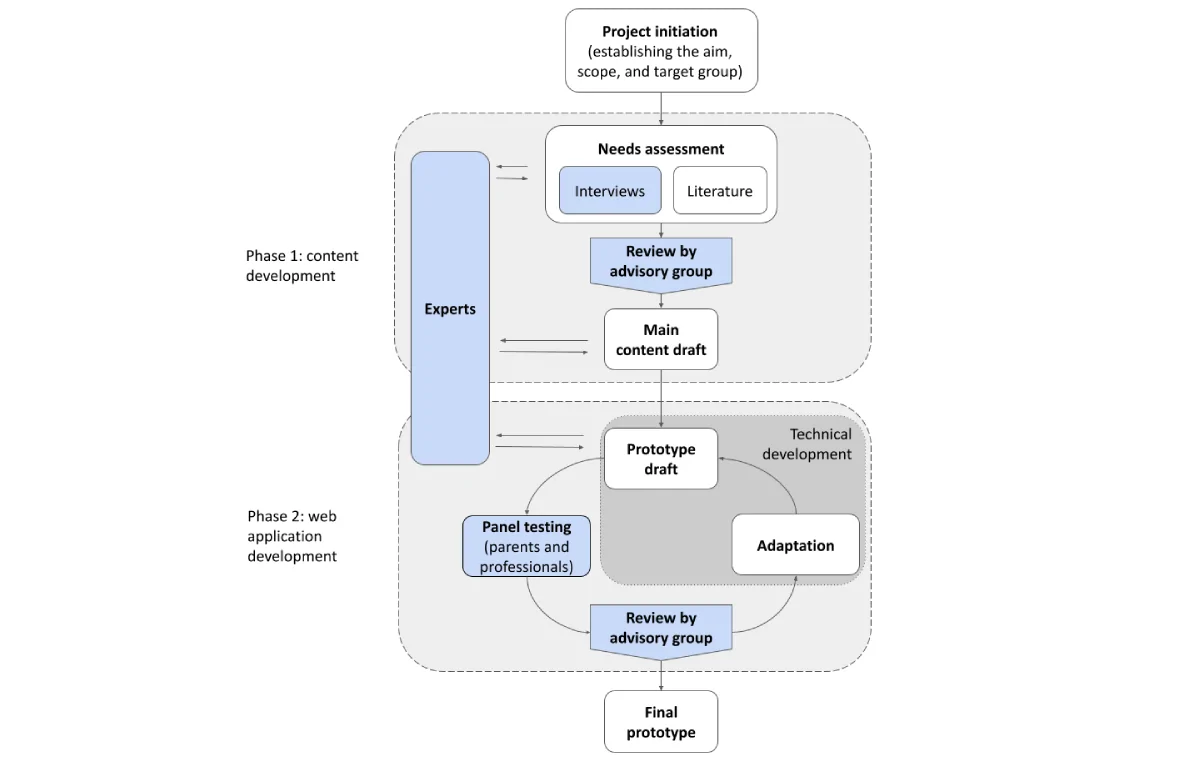
Flowchart of the development process. Steps involving active stakeholder participation are highlighted in blue.

The process was guided by systematic development protocols for DAs [25,26] and involved two main phases: (1) drafting the main content and (2) iterative web application development. Our user-centered approach involved engaging stakeholders across different phases. The flowchart in Figure 1 provides an overview of the phases and the involvement of stakeholders.

We formed an advisory group of stakeholders to provide guidance and input throughout the development process. On the basis of the recommendations of Elwyn et al [26], we aimed for a stakeholder group of 6 to 10 members with balanced representation across our stakeholder groups. These groups included the target group (parents), CYH professionals providing vaccination services, medical advisers from the NIP hosted by the National Institute of Public Health, and experts in communication and DA development. Additionally, subject matter experts were consulted on an ad hoc basis whenever specialized expertise was needed. The stakeholder group members and experts were purposefully selected through the research team’s professional network.

#### Phase 1: Content Development

During the first phase, we based the content of the PDA on the results of a needs assessment. This consisted of a review of relevant literature and interviews to identify parents’ questions, information needs, and decision-making needs. We searched for relevant literature in the PubMed database using the keywords “childhood vaccination,” “information needs,” “decision-making needs,” and “parents” and searched for additional literature using the snowball method. After reviewing the search results, we prioritized key papers that contained relevant information and were applicable to our setting. We extracted the described information and decision-making needs.

Additionally, we conducted brief informal interviews with parents visiting a CYH clinic to explore their information and decision-making needs. Parents were approached at 2 different clinics before their appointment and received verbal information about participation. When parents agreed, a brief informal interview was conducted in person at the respective clinic by a member of the research team (DB-V) and took 10 to 20 minutes. During the interviews, notes were taken, no recording was made, and no personal identifying details were collected. On the basis of the notes, parental needs were summarized, and interviews were continued until no new topics came up. The findings from the literature and interviews were triangulated with a CYH clinician who was highly experienced in counseling parents with vaccine hesitancy. During the triangulation process, the identified needs were grouped into main topics and prioritized. We chose this approach because we aimed to provide a concise and clear overview of the most important topics and to cover the main needs rather than answer every detailed need. Some needs were highly individual and competed with those of other individuals, and integrating these would compromise the accessibility and readability of the DA. The main topics were subsequently reviewed by the advisory group and then used to inform the first draft of the PDA’s content. We also involved a health literacy expert and a CYH clinician as subject matter experts to thoroughly review the first version of the PDA draft; they were both recruited through the research team’s professional network.

#### Phase 2: Iterative Web Application Development

In the second phase, a company was contracted to undertake the technical realization of the draft content into a prototype web application. As we used a software template previously developed for a vaccination DA, we collaborated with the same design company and its user experience (UX) professional and could thereby benefit from their expertise in this field.

The prototype was evaluated by panels of both parents and professionals and adapted in iterative cycles. We used different routes to engage panelists as we sought to include a variety of perspectives. We invited professionals involved in routine childhood vaccination care through a newsletter call at 2 different regional CYH organizations and selected both physicians and nurses. Additionally, we invited parents through a call in a childcare service newsletter and through collaboration with an existing low socioeconomic parent panel [27].

First, we focused on general use and navigation through the app and invited a broad group of panelists to conduct a robust evaluation of the prototype. Panelists reviewed the prototype individually online via Microsoft Teams, with a member of the research team taking notes. These sessions took approximately 30 to 40 minutes. In this cycle, we verbally administered the System Usability Scale (SUS) to assess the level of usability [28]. We analyzed session notes to identify issues with the PDA’s design, content, and structure and discussed them within the research team. Additional review sessions with new panelists were planned until no new issues were identified. On the basis of the panelists’ experiences with the prototype, we formulated adaptations to improve the design, structure, and content of the PDA. These were subsequently discussed with the advisory group and implemented. From the second cycle of review sessions onward, we focused on fine-tuning the suitability of the prototype for users with lower health literacy skills. The prototype was evaluated in person at a more detailed level with members from the low socioeconomic parent panel who had low or medium levels of educational attainment. These sessions took approximately 60 minutes. Similar to the previous cycle, we analyzed session notes, recommended adaptations, and reviewed the adaptations with the advisory group before implementation. We planned a final round of test sessions to evaluate if the adaptations had the expected result.

### Ethical Considerations

This project was carried out in accordance with institutional and national guidelines. The consulted stakeholders gave verbal consent to participate and, as compensation, they received a gift voucher. No personal identifying details were collected. We described the methods for the development of the DA as part of a larger project that also included a feasibility study. This study protocol was submitted to our ethics review board and deemed exempt from full review (2024-17606).

## Results

### Phase 1: Content Development

#### Needs Assessment

We assessed the available literature from 2012 to 2022 addressing the needs of parents when making decisions about childhood vaccination. Additionally, interviews with 18 parents conducted between May and June 2023, along with triangulation with a CYH clinician, confirmed and added to the findings of the literature assessment.

We grouped the identified needs into the 5 topics shown in Textbox 1: disease descriptions, vaccination schedule, practical information, factual, balanced, reliable information, and discussion and deliberation.

**Parental needs identified during the needs assessment.**

**Disease descriptions**

Parents expressed the need to receive information about the diseases that vaccinations aim to prevent, including the risk of contracting these diseases, the necessity of vaccination, and the effectiveness, side effects, and safety of the vaccine itself [29-31]. Most parents indicated that their baseline knowledge was limited. This was not necessarily experienced as problematic, and preferences for the level of detail varied greatly, ranging from highlighting key information to requesting an extensive representation of risks.

**Vaccination schedule**

Parents also indicated a need for information about the timing and rationale of the vaccination schedule. This included an explanation for combining multiple vaccines and the need for administering multiple doses of the same vaccine at different points in time [29].

**Practical information**

Parents requested information about the administration of vaccines, including the injection site and care for children after vaccination [29]. They also requested practical guidance on how to reduce discomfort after vaccination.

**Factual, balanced, reliable information**

Parents expressed the need to receive balanced, accurate, reliable information in a neutral tone, written in an understandable manner, preferably from independent sources, and presented in a nondirective manner [29,32]. Some parents also indicated that, to achieve the right balance, details about severe disease outcomes should be left out.

**Discussion and deliberation**

Parents indicated the need to deliberate on and discuss the decision with others, such as health care professionals, relatives, or friends [32-34]. Additionally, parents wanted to hear the views and decisions of other parents.

#### Review by the Advisory Group

The findings of the needs assessment were reviewed by the advisory group. This group was permanently composed of 7 members, including experts, parents, CYH professionals, and representatives of the National Institute of Public Health. This group endorsed the guiding principles and identified needs but did not support the request to omit details of illness that can be prevented by vaccination. Members also wanted to include upcoming changes to the NIP and recommended referring parents to health care professionals and/or their CYH centers if they had questions after using the PDA.

#### Main Content Draft of the PDA

We based the main content of the PDA on the identified parental needs, evidence-based information about vaccination, and how to communicate about vaccination. We designed 2 modules. One module provided information to support an informed choice, and the other module promoted reflection on values and the views of other parents to support a deliberate choice. The initial draft covered all 5 topics identified in the needs assessment (Textbox 1). In November 2023, a health literacy expert suggested readability improvements, a reduction in the volume of information, and visual elements to support the text. At the same time, the content was reviewed by a CYH clinician for accuracy, tone, and alignment with practice. Table 1 shows illustrative examples of adaptations following their advice.

**Table 1:** Illustrative examples of adaptations to the PDA based on expert consultation.

Expertise and advice			Adaptations
**Health literacy**			
	Use active voice consistently	Changed “Children receive vaccinations to protect them from illness” (passive voice) to “Vaccinations protect children from illness” (active voice)	
	Avoid complex sentence constructions and abstract words	Changed “Vaccination contributes to public health” to “vaccination helps keep people healthy” Changed “A vaccine protects against a combination of multiple diseases at the same time” to “a vaccine protects against multiple diseases” Changed “airways” to “lungs”	
	Explain the purpose of each text section	Added the following to the introduction of the decision support module: “Have you read enough information about vaccinations for your child? Continue here to organize your thoughts. At the end, you will get an overview of what is important to you.”	
**CYH^a^**			
	Align terminology to avoid confusion	Referred to the “National Immunization Program”	
	Consider including a reference to other tools used in practice	Included a reference to a tool parents can use to chat with a CYH nurse	
	Include practical information about vaccination appointments	Added information about vaccination invitations and routine vaccination services	
**User experience**			
	Limit the number of levels in the tool that users can navigate through	Restructured the design to three main layers: (1) welcome page, (2) modules page, and (3) specific information page or feature	
	Use a professional yet warm and calm style	Placed more detailed information in drop-down panels Selected a color palette and main images	

^a^CYH: child and youth health care.

A significant challenge was how best to present probabilities. A general recommendation for DAs is to provide numerical information in a consistent format [35]. This was difficult for the childhood vaccination PDA, as the range of probabilities varies for each of the 12 diseases and depends on many other factors, such as overall population vaccination coverage and the child’s age. Presenting probabilities using scenarios of best, worst, and most likely outcomes was a possible solution [36]. In the first draft of the PDA, we included both numerical and scenario-based information so that we could seek guidance from our stakeholders.

### Phase 2: Iterative Web Application Development

#### Design and Technical Development of the First DA Prototype Draft

The hired company created a prototype, and a UX professional supported us in designing a layout that was appealing, professional, user-friendly, and easy to navigate. Illustrative examples of subsequent adjustments are shown in Table 1. Additionally, we added a read-aloud feature to support better text comprehension.

#### Iterative Cycles of Panel Testing, Review by the Advisory Group, and Adaptations

We subjected the prototype draft to iterative cycles of testing, review, and adaptation (Figure 1). During the first cycle, we focused on evaluating usability and content through online testing sessions with panelists, including parents and professionals. We observed how they used the prototype through screen sharing and encouraged them to think aloud. This gave us insights into how users navigated the PDA. Their overall impression was positive. They found the scenario-based format of risk representation (best-case and worst-case scenarios) clear and concise, although some preferred numerical risks. Panelists also found the overview of considerations provided in the decision support module helpful.

The level of usability according to the SUS ranged from 68 to 93 (out of 100), with an average of 78, indicating good usability [28]. In subsequent test cycles, we focused on parents with lower health literacy skills. We evaluated their UX using in-person test sessions and observed how they used the prototype. These parents found some words confusing, and we made changes to address this issue.

After each cycle, we discussed adaptations with the advisory group. Table 2 shows the characteristics of the panels, illustrative examples of comments and feedback provided by the panels, and subsequent adaptations made in each cycle. A key editorial decision involved reducing the amount of information, categorizing the information into essential and additional topics (refer to Final PDA Prototype section), and simplifying the navigation within the decision support module.

**Table 2:** Illustrative examples of adaptations to the PDA based on panel consultation.

Cycles, panel characteristics, and comments				Adaptations
**Cycle 1^a^**				
	**General**			
		“It is not clear which organization has created and who made this decision aid.”	We improved logo visibility and added information about the team that created the DA in the “About us” section.	
		“Overall, this is a lot of information.”	We critically reviewed the topics covered by the DA and prioritized information needed for decision-making. For example, practical information on the administration of vaccines was removed.	
		“The large amount of text makes it difficult and also a bit boring. Pictures, videos, and a personal story are missing.”	We made adaptations to reduce the length of text blocks and replaced text with visual elements, including infographics and videos. For example, educational videos and a video with a clinician sharing experiences with parents’ vaccination decision-making were added.	
		“The social aspect of vaccination is a bit forced here, this is really too much for me. I prefer the DA to focus on my personal situation.”	As it was an important principle to convey the societal aspects of vaccination, we did not make any adaptations.	
	**Information module**			
		“The info on risks and safety is limited and does not address concerns that parents may have.”	Information on this topic was spread over several tiles. We merged and consolidated this information under 1 tile.	
		“The info about diseases is quite a list and not inviting to read.”	We restructured this information, grouped diseases by vaccine, and placed text blocks in drop-down sections.	
	**Decision support module**			
		“The statements are difficult to read.”	The wording and visual representation of the statements were improved.	
		“Disappointing that the [value clarification] exercise only gives an overview of what is important in my decision, but does not give advice based on my input.”	It is neither feasible nor desirable to generate tailored advice based on user input, as the advice for every parent is to vaccinate their child. The exercise aims to clarify which values are important to parents in their decision-making. We adjusted the wording at the start of the module to manage expectations. At the end of the exercise, we explicitly advised users to discuss the results with a health care professional.	
**Cycle 2^b^**				
	**General**			
		“The links to a different website are confusing, I don’t like to be directed to another website.”	We reduced the number of links directing users to other websites. Where such links were still present, this was explicitly indicated to simplify navigation.	
		“The information on how vaccines work is not clear. It now seems that children make antibodies against diseases even without vaccination. I now think that vaccines are not needed.”	The information on how vaccines work was simplified and adjusted to capture the main message.	
		“The information on how to find reliable information is not helpful. I expect to find examples of other reliable sources.”	We adopted this suggestion and presented links to key websites with reliable information.	
**Cycle 3^c^**				
	**General**			
		“The information on the different diseases that children are vaccinated against, gives a nice and good overview. I would like a bit more background information on how common these diseases are elsewhere in the world.”	On the basis of previous feedback that the information was too much and the subsequent reduction of content, we decided not to include additional background information on diseases.	

^a^n=13; 9 parents (mothers of children aged ≤4 years) with a high level of educational attainment and 4 CYH professionals involved in vaccination care for children aged ≤4 years (1 physician and 3 nurses) from 2 CYH organizations.

^b^n=4; 4 parents with lower educational attainment.

^c^n=3; 3 parents with lower educational attainment.

### Final PDA Prototype

#### Overview

We considered the prototype final when the panel sessions did not yield any new feedback and when users could navigate the PDA easily. The Dutch Foundation for Easy Reading assessed the final content and awarded a certificate recognizing its use of plain and ordinary Dutch. The final PDA prototype consists of the following components.

#### Welcome Page

**Figure 2:**
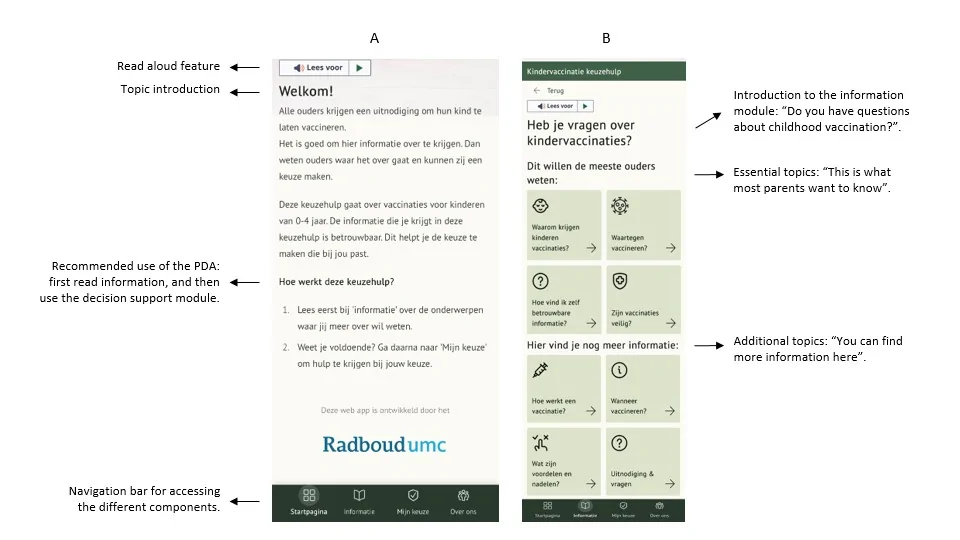
Screenshots of the welcome page (A) and information module (B). The arrows indicate the main elements of the PDA. The screenshots were taken using a mobile display of the PDA, images are not shown due to potential copyright concerns. The full translation of the text shown in this figure is provided in the multimedia appendix.

The PDA begins with a welcome page that explains what the PDA is, who it is intended for, and how to use it (Figure 2A). From the navigation bar, users can navigate to the different components.

#### Information Module

This module features a tiled architecture that organizes topics into 2 categories: essential information titled “This is what most parents want to know” and additional information titled “You can find more information here” (Figure 2B). Each tile links to a question and its answer, presented in the form of text and/or visual information. The essential topics cover the individual and societal aspects of vaccination, the diseases that vaccines are designed to prevent, how to find reliable information, and vaccine safety. The additional topics include how vaccines work, the vaccination schedule, the advantages and disadvantages of vaccination, and details about the vaccine invitation.

#### Decision Support Module “My Choice”

**Figure 3:**
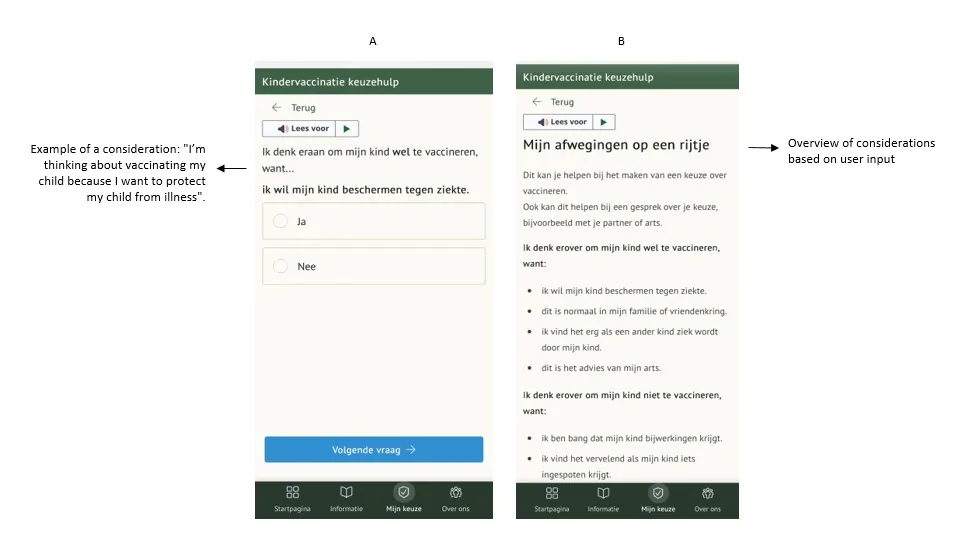
Screenshots of the value clarification exercise in the decision support module, showing (A) an example of a consideration and (B) an overview of considerations relevant to the user, presented at the end of the exercise. The screenshots were taken using a mobile display of the PDA, images are not shown due to potential copyright concerns. The full translation of the text shown in this figure is provided in the multimedia appendix.

The main page of the decision support module features an explanation of the different steps in the decision-making process and a video of a clinician’s experience in assisting parents through this process. This page links to “The choice of others,” with perspectives of other parents on vaccination, along with an icon array visualizing vaccination coverage. Within this module, users can also access the value clarification exercise (“My Choice”). This exercise allows users to reflect on their values and considerations related to their vaccination decision. Users are invited to agree or disagree with various considerations regarding their vaccination decision and can also add their own. Figure 3A shows an example of a consideration. At the end of the exercise, a customized overview of considerations relevant to each user is generated (Figure 3B). This overview can serve as a basis for a conversation with a health care professional.

#### About Us Page

To clarify who designed the PDA, we included an information page that introduces the project team members and briefly explains the evidence on which the PDA is based.

## Discussion

### Principal Findings

We co-developed a PDA that addresses both the individual and societal aspects of childhood vaccination within the Dutch immunization program. In doing so, we aimed to address cognitive, emotional, and societal aspects of vaccine hesitancy [6]. Parents who participated in the co-development process expressed that they felt this PDA was needed and emphasized that it should be tailored to meet the needs of parents with lower levels of educational attainment.

Our user-centered approach is the main strength of this project. This allowed us to tailor the PDA to the needs of our target group, a critical step in developing potentially effective interventions that address hesitancy [37]. We prioritized the interests of hesitant parents and those with lower health literacy, who probably will benefit most from a decisional support intervention [16,38]. For example, parents with lower levels of educational attainment had difficulty understanding the key message about how vaccines work (refer to cycle 2 described in Table 2). We subsequently made adjustments to improve clarity and evaluated them in the next cycle. Another strength is that we used a systematic and cyclical approach, combining various methods (expert consultation, user evaluation, and readability assessment) to ensure the suitability of the PDA in practice. In addition, in line with our guiding principles, we combined elements of risk communication (“Information” module) with value clarification (“My Choice” module), which are more likely to be effective in supporting those who are hesitant than information alone [39].

A limitation of this project is that we developed the PDA in Dutch. Professionals and parents requested that the PDA be made available in other languages as well. It is important to explore if this would increase its future reach within the Dutch population. Another limitation is that we were not able to address all needs. For example, some parents preferred very detailed disease-specific information (refer to cycle 3 described in Table 2). This conflicted with our focus on vaccination as a general concept and our prioritization of the needs of parents with lower health literacy. Consequently, we opted for short text blocks alternating with visual elements instead of extensive written information. This indicates that a PDA alone will not satisfy all users’ needs and should be used alongside other interventions, such as educational materials and conversations with health care professionals. Another limitation is that we could have involved a more diverse group of stakeholders in this project. When we evaluated the PDA with parents, we asked them to reflect on the period when they were pregnant but did not include prospective parents during pregnancy in our panel evaluations. We could also have involved our stakeholders more, for instance, when designing the structure and layout of the PDA. Although we mitigated this by evaluating these aspects with panelists afterward, we recommend a diverse group of stakeholders to participate actively in each development step.

### SDM and the Role of the PDA

When developing this PDA, we considered the guidelines and criteria of the International Patient Decision Aid Standards (IPDAS) Collaboration [12]. These guidelines emphasize a balanced representation of the risks and benefits of different options for the individual. However, in the case of vaccination, individual choices also have a wider societal impact through group immunity. Therefore, we considered it vital to address the societal aspect of vaccination in our PDA. Some panelists experienced this as coercive and favored focusing only on their individual perspective (refer to cycle 1 described in Table 2). As this was clearly contrary to our principles, we did not adopt this suggestion. This illustrates that although stakeholder input guided the development, it was subject to certain limits. This also illustrates how we adapted the traditional SDM approach to fit decision-making about routine childhood vaccination. Alternative criteria are needed to guide the development of DAs to support similar evidence-guided decision-making in a public health context. When carefully adapted, an SDM approach could contribute to patient engagement, support informed decision-making, and foster dialogue with health care professionals, even in a public health context.

Supporting decision-making about childhood vaccination warrants a complex balance between strong evidence-based recommendations for accepting vaccination and its population-level benefits, while also considering benefits, harms, and preferences at the individual level. This means allowing space for individual concerns and considerations without compromising the scientific evidence supporting population-level interventions. Throughout the development of this PDA, we carefully sought this balance by (1) providing evidence-based information in an accessible format to help hesitant parents navigate their way through the vaccination information landscape and (2) providing decisional support, allowing parents to inventorize their individual values and considerations as input for further conversation with a health care professional.

### Conclusions

Interventions aimed at addressing vaccine hesitancy have primarily concentrated on mass-media communication and on interactions between parents and health care providers. By introducing this PDA about childhood vaccination, prospective parents and parents can use it in a way and at a time that best suits their decision-making process. Future research should focus on the best timing for using this PDA to support informed and deliberate decisions. As more vaccines are being added to the NIP at a very young age and vaccination decisions are likely to start before childbirth [18], we recommend evaluating this PDA from pregnancy onward.

## Data Availability

The data supporting the development of the DA are included in this published article. The needs assessment and advisory group meetings were not transcribed and therefore no raw data are available.

## Abbreviations

CYH: child and youth health care
DA: decision aid
GUIDED: Guidance for the Reporting of Intervention Development
IPDAS: International Patient Decision Aid Standards
NIP: National Immunization Program
PDA: parent-facing decision aid
SDM: shared decision-making
SUS: System Usability Scale
UX: user experience
WHO: World Health Organization
